# Characteristics of taste dysfunction in COVID-19 subjects coming from two different countries

**DOI:** 10.1007/s13365-021-00942-8

**Published:** 2021-03-22

**Authors:** Alfonso Luca Pendolino, Giancarlo Ottaviano, Bruno Scarpa, Annamaria Cattelan, Julie A. Andrews, Peter J. Andrews

**Affiliations:** 1 Department of Ear, Nose and Throat, Royal National ENT & Eastman Dental Hospitals, London, UK; 2 Ear Institute, UCL, London, UK; 3 Department of Neurosciences, Otolaryngology Section, University of Padova, Padova, Italy; 4 Department of Statistical Sciences and Department of Mathematics Tullio Levi-Civita, University of Padova, Padova, Italy; 5 Unit of Infectious Disease, University Hospital of Padova, Padova, Italy; 6 Department of Microbiology, Whittington Health, London, UK

**Keywords:** COVID-19, Rhinology, Taste, Taste dysfunction, Survey

## Abstract

Taste dysfunction (TD) has been recognised, together with olfactory dysfunction, as a key presenting symptom of COVID-19. The capability to recognise flavours, flavour intensities and aroma characteristics can be highly variable within the same population, as well as potentially diverse between culturally different populations. The aims of this study are to evaluate whether a difference in the types of TD presentation amongst COVID-19 positive subjects can be demonstrated and whether a difference exists between populations of different cultures.

Taste dysfunction (TD) has been recognised, together with olfactory dysfunction, as a key presenting symptom of COVID-19, and its prevalence has been estimated to be as high as 30.4% in the general population (Bartheld et al. 2020). Most cases of TD are associated with OD suggesting that gustatory alteration is usually linked to an impairment of retronasal olfaction rather than impairment of gustation itself (Whitcroft and Hummel 2020). Nonetheless, the angiotensin-converting enzyme 2 receptor, which is the main host cell receptor of SARS-CoV-2, is widely expressed on oral mucosa epithelial cells (Xu et al. 2020) and this may explain the taste impairment experienced in the early stage of COVID-19 as well as the more rare cases of isolated TD (Bartheld et al. 2020).

The capability to recognise flavours, flavour intensities and aroma characteristics can be highly variable within the same population (Doty 2019), as well as potentially diverse between culturally different populations. Certain populations may be more prone to detect a TD if a particular flavour which they have been accustomed to has been affected (Sjöstrand et al. 2020).

To the best of our knowledge, characteristics of TD presentation amongst COVID-19 positive subjects have not been evaluated and the differences in TD presentation between culturally different populations have not been assessed. The aims of this study are to evaluate whether a difference in the types of TD presentation amongst COVID-19 positive subjects can be demonstrated and whether a difference exists between populations of different cultures.

Between May 26 and June 10, 2020, we conducted a survey in two European hospitals (London (UK) and Padua (Italy)) on COVID-19 positive subjects who complained of olfactory and/or taste dysfunction during the course of their disease (Andrews et al. 2020). The recipients of this survey were mild-to-moderate symptomatic COVID-19 positive health-care workers who tested positive by RT-PCR for SARS-CoV-2 and were working at their own hospital during COVID-19 pandemic. Participants were selected using the databases of the Microbiology Laboratory in London and the Infectious Disease Department in Padua. The study was approved by the UCL Research Ethic Committees (IRAS:156,511), the UCL joint research office and the Padua Otolaryngology Section’s in-house ethical committee (n 056881). The survey questionnaire was validated by both ENT and infection clinicians as well as patient advocates to ensure clarity and to exclude ambiguity. All respondents were invited to take part in this survey via email which included a study information pack and a consent form. Those who accepted and signed the consent form received the questionnaire via email in London and by hand in Padua. Inclusion criteria were age > 18 years old, laboratory confirmation of SARS-CoV-2 infection, subjects with mild-to-moderate symptoms (defined as home-managed subjects with symptoms not requiring intensive care or hospital admission), good comprehension of the language used in the questionnaire and absence of any clinical impairment to complete the questionnaire. For the specific aim of the study, only those participants with a reported taste impairment at the moment of questionnaire administration were considered. Subjects with a past history of TD or those whose sense of taste had completely recovered at the moment of the survey were excluded.

Impairment of the four main flavours in addition to water, which has recently been shown to be tasted as a separate flavour (Sjöstrand et al. 2020; Rimmer et al. 2019), was investigated. Specifically, the participants had to mark which of the listed flavours they were no longer able to taste. Furthermore, they were asked if they had a distortion of the taste (dysgeusia), namely if they still continued to detect the different flavours but distorted. We excluded the flavour ‘umami’ because of its significant variability in recognition amongst different populations (Sjöstrand et al. 2020). Quantitative variables were compared using the Wilcoxon test while binary variables using the Fisher’s exact test. The heterogeneity within samples was tested with pairwise comparison of prevalence adjusted by the Holm correction. The chi-square test was adopted to compare the frequencies of the numbers of flavours affected within samples.

One-hundred and fifty-five HCWs, 119 from London and 36 from Padua, received the questionnaire. The different method of questionnaire administration led to a different response rate of 70.6% (84/119) in London and of 100% (36/36) in Padua. After further analysis, we excluded 2 participants who did not accept the consent form and 4 participants who did not answer any question, leading to a final population of 114 HCWs who completed the initial survey. Among them, only 47 subjects, 23 Londoners and 24 Paduans, were still complaining of TD when questionnaire was administered and were finally considered for the analysis. The majority of the participants were female, middle-aged, white and reported TD to be associated with OD (Table 1). When comparing the two hospitals, a statistically significant difference was observed in the reported affected flavours (sour and bitter higher among Londoners) and the presence of distortion of taste (dysgeusia higher among Paduans). Additionally, a statistically significant difference in the ethnical composition was noted when comparing the two samples. Apart from a significantly higher rate of white subjects in the London group and of dysgeusia reported by the Italian participants, no other, statistically significant differences were found when looking at the two populations separately (Table 1).

**Table 1:** Characteristics of taste dysfunction in the two populations

	London (*n* = 23)	Padua (*n* = 24)	*p* value (within hospital)		*p* value (between hospitals)
			London	Padua	
Age, median (P25–P75) (year)	39 (33–48)	36 (27.5–44.5)	-	-	0.16
Sex, no (%)					
Female	17 (73.9%)	19 (79.2%)	-	-	0.74
Male	6 (26.1%)	5 (20.8%)			
Ethnicity, no (%)^a^					
White	12 (57.1%)	24 (100%)	0.02*	-	0.002*
Asian	7 (33.3%)	0 (0.0%)			
Black/African/Caribbean	2 (9.6%)	0 (0.0%)			
Mixed/multiple ethnic groups	0 (0.0%)	0 (0.0%)			
Missing	2	0			
Smell dysfunction, no (%)					
Yes	22 (95.7%)	24 (100%)	-	-	0.45
No	1 (4.3%)	0 (0.0%)			
Type of flavour affected, no (%)					
Sweet	12 (52.2%)	7 (29.2%)	1^b^	1^b^	0.23
Sour	14 (60.9%)	5 (20.8%)			0.016*
Bitter	15 (65.2%)	3 (12.5%)			0.001*
Salty	11 (47.8%)	4 (16.7%)			0.06
Water	8 (34.8%)	7 (29.2%)			1
Numbers of flavours affected, no (%)^a^					
None (only dysgeusia)	4 (17.4%)	14 (60.8%)	0.88	0.001*	0.065
One	2 (8.7%)	2 (8.7%)			
Two	4 (17.4%)	2 (8.7%)			
Three	5 (21.7%)	1 (4.4%)			
Four	5 (21.7%)	3 (13.0%)			
All of them	3 (13.1%)	1 (4.4%)			
Missing	0	1			
Dysgeusia, no (%)					
Yes	9 (39.1%)	21 (91.3%)	-	-	0.001*
No	14 (60.9%)	2 (8.7%)			
Missing	0	1			

^*^Significant *p* values marked in italics. Level of significance *p* < 0.05

^a^Valid percent, not including missing values

^b^The same *p* value (*p* = 1) was obtained when comparing each couple of affected flavours, apart from the comparison between bitter and water (*p* = 0.77) in the London group

A difference in the presentation of TD between the London and Padua populations was observed with respect to flavour type being affected and dysgeusia. Particularly, a loss in the flavour of sour and bitter was clearly reported by Londoners, whereas the Italian participants reported a significantly higher rate of taste distortion (dysgeusia). When looking at the single populations separately, it would appear the London population has a more severe TD with a higher number of flavour types being affected compared with the Paduan population. However, we did not show that a specific flavour or number of flavours being affected occurred more commonly as a result of SARS-CoV-2 infection (*p* = 1).

Our results demonstrate a statistically significant disparity in taste perception between the two countries which may be due to genetic predisposition, cultural learning or a difference threshold in the taste perception which could explain the higher rate of dysgeusia amongst Italians. Importantly, the London population was a more ethnically diverse population which may be a confounding factor. Our data also confirms previous findings that TD is more frequent amongst middle aged and female subjects and generally associated with an OD (Bartheld et al. 2020).

A limit of the study was that TD was not confirmed through psychophysical tests (Rimmer et al. 2019). However, during the present pandemic, psychophysical tests have not been available or fully feasible in many countries and, therefore, we believe that in an emergency condition self-rated symptoms remain of value.

Taken together, our results confirm that a cultural variability in taste appreciation or perception should be borne in mind when investigating subjective TD amongst COVID-19 positive subjects especially when it is considered a key symptom in COVID-19 presentation. In the future, it would be interesting to study if taste differences exist between the two countries by including healthy subjects not complaining of any TD, and, possibly, using taste strips.
